# Different Strategies for the Treatment of Age-Related Macular Degeneration in China: An Economic Evaluation

**DOI:** 10.1155/2016/7689862

**Published:** 2016-04-20

**Authors:** Bin Wu, Jin Li, Houwen Lin, Haixiang Wu

**Affiliations:** ^1^Medical Decision and Economic Group, Department of Pharmacy, Ren Ji Hospital, South Campus, School of Medicine, Shanghai Jiaotong University, Shanghai 201114, China; ^2^Department of Ophthalmology, Ren Ji Hospital, School of Medicine, Shanghai Jiaotong University, Shanghai 200127, China; ^3^Department of Ophthalmology, Eye & ENT Hospital, Fudan University, Shanghai 200031, China; ^4^Shanghai Key Laboratory of Visual Impairment and Restoration, Eye & ENT Hospital, Fudan University, Shanghai 200031, China

## Abstract

*Purpose.* To assess the cost-effectiveness of bevacizumab compared to ranibizumab, verteporfin photodynamic therapy (PDT), and usual care for the treatment of age-related macular degeneration (AMD) in China.* Methods.* A Markov model was developed according to patient visual acuity (VA) in the better-seeing eye (Snellen scale). Four cohorts of patients were treated with one of the following therapies: bevacizumab, ranibizumab, PDT, or usual care. Clinical data related to treatments were obtained from published randomized clinical trials. Direct medical costs and resource utilization in the Chinese health care setting were taken into account. Health and economic outcomes were evaluated over a lifetime horizon. Sensitivity analyses were performed.* Results.* Treatment with ranibizumab provided the greatest gains in quality-adjusted life-years (QALYs). The cost per marginal QALY gained with bevacizumab over usual care was $1,258, $3,803, and $2,066 for the predominantly classic, minimally classic, and occult lesions, respectively. One-way sensitivity analysis showed considerably influential factors, such as utility values and effectiveness data. Probabilistic sensitivity analysis indicated that, compared to usual care, PDT and ranibizumab most cases would be cost-effective in the bevacizumab arm at a threshold of $7,480/QALY.* Conclusion.* Bevacizumab can be a cost-effective option for the treatment of AMD in the Chinese setting.

## 1. Introduction

Age-related macular degeneration (AMD) is a progressive chronic macular disease of the central retina that leads to vision loss and significant functional impairment worldwide [1]. In the white population aged 40 years and older, the prevalence of early and late AMD is nearly 6.8% and 1.5%, respectively [2]. The age-specific prevalence of late AMD in Asians was largely similar to that in white people [3]. Age is one of the main risk factors for AMD; according to a report by the World Health Organization (WHO), the estimated number of cases will double by 2020 due to trends in the growth of aging populations [4]. In China, the prevalence of AMD in the early 1990s was nearly 5%, and over the last decade, the prevalence has increased nearly twofold in the aging population. At present, blindness caused by AMD has become one of the most important health challenges in China [5].

Most visual loss occurs in the late stages of AMD in one of two forms: geographic atrophy (“late dry”) or neovascular (“wet”) AMD. Although only an estimated 10%–15% of all AMD cases take the neovascular form, it is responsible for more than 80% of severe visual loss (visual acuity of 20/200 or worse) due to choroidal neovascularization, which results in hemorrhage and fluid leakage and fibrosis [6]. Based on the pattern of lesions, wet AMD can be classified into three subtypes: occult (35%–73% of patients), minimally classic (35%), and predominantly classic (20%–44%) [7–9]. Several biological mechanisms are thought to be involved in the pathogenesis of AMD, particularly vascular endothelial growth factor (VEGF). VEGF is a key regulator of angiogenesis, and overexpression can block vascular growth and neovascular regression [10]. In last decade, new agents targeted against VEGF, such as bevacizumab (Avastin, Genentech/Roche) and ranibizumab (Lucentis, Genentech/Novartis), have substantially changed the patterns of therapies for wet AMD. They have been shown to be superior to conventional treatments (e.g., photodynamic therapy [PDT]) in improving visual acuity gain and preventing visual acuity loss in patients with wet AMD [5, 6, 11–15].

These new agents have improved clinical outcomes as well as reducing the human and socioeconomic consequences of AMD. Visual impairment can result in substantial functional loss, reduced quality of life, and increased risk of comorbidities, such as depression and falls, which can increase the socioeconomic burden on patients and societies [16–18]. Evidence from US medical claims data shows that the total annual direct cost of AMD is 575–733 million dollars [10]. In Western Europe, the yearly budgetary impact of AMD in 2002 was from €51.3 to €101.1 million [19]. There are a number of published economic analyses comparing different therapies for AMD, including laser photocoagulation, verteporfin with PDT, pegaptanib, bevacizumab, and ranibizumab, which show that these therapies have improved health outcomes and reduced resource consumption associated with visual impairment [20]. However, no published studies have investigated the cost-effectiveness of these therapies in a health resource-limited setting.

Verteporfin and ranibizumab have been licensed for AMD treatment in China. However, these agents are not covered by medical insurance. The higher cost of these two agents has limited their widespread use in Chinese clinical practice. At present, bevacizumab has also been approved to treat cancers. However, its similar efficacy and much lower cost than ranibizumab have stimulated the off-label use of bevacizumab for wet AMD [5]. The objective of this study was to evaluate the cost-effectiveness of bevacizumab, ranibizumab, and verteporfin with PDT versus usual care for the predominantly classic, minimally classic, and occult AMD populations. The usual care in the current analysis is the supportive care based on Chinese ophthalmologist's opinion, which would annually include two outpatients' visit for an ophthalmologist with one optical coherence tomography. The perspective of the Chinese health care system, representative of a health resource-limited setting, was adopted in this analysis, and only direct costs were considered.

## 2. Methods

### 2.1. Model Overview

**Figure 1:**
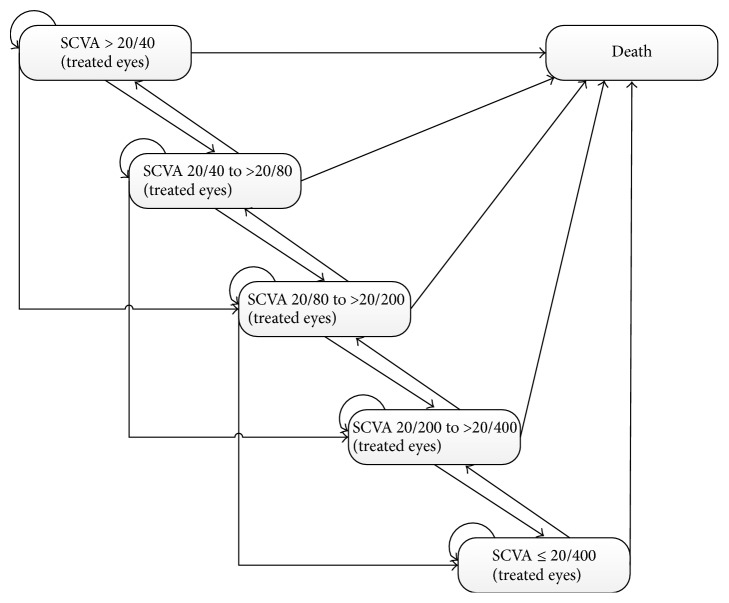
Overview of the Markov model structure. Health states are defined by Snellen chart visual acuity (SCVA) in the treated eye. Patients have a risk of death at any state in the model.

A Markov model was used to simulate the lifetime disease course of wet AMD for a cohort of patients. The model structure is shown in Figure 1, including the death state and the five health states based on the Snellen chart of visual acuity (SCVA) in the better-seeing eye as follows: SCVA >20/40, 20/40 to >20/80, 20/80 to >20/200, 20/200 to >20/400, and ≤20/400 (light perception only). Transitions between the health states can occur every 3 months according to specified probabilities. After each cycle, the SCVA could remain in the current state, improve by at least 3 lines, or worsen by 3 to 6 lines and >6 lines. Patients could transition to the death state from any health state in the model. The R statistical environment (version 3.2.2; R Development Core Team, Vienna, Austria) was used to develop and solve the model.

The baseline characteristics of the hypothetical cohorts are based on a previously published study [21]. All patients were newly diagnosed with wet AMD and the starting age for treatment at the base case was 73.6 years. Due to a lack of China-specific data, the initial SCVA was derived from a previously published study based on a Chinese patient cohort [22]. Patients would receive one of four of the following treatments: bevacizumab, ranibizumab, PDT with verteporfin, or usual care. In the model, active treatments were assumed to take up to 2 years.

The cost and health outcome were measured for each treatment in patients with wet AMD; the quality-adjusted life-year (QALY) gained was used as the primary health outcome. A cost-utility analysis was conducted and the incremental cost-effectiveness ratio (ICER) was estimated by comparing two treatments. Based on a WHO recommendation, the per capita gross domestic product (GDP) of China per QALY was used as a cost-effectiveness threshold in this analysis [23–25].

In the current analysis, a yearly discount rate of 3% was applied to both costs and QALYs, which is a general approach in the Chinese setting.

### 2.2. Clinical Efficacy

The clinical data for each AMD treatment were taken from published clinical trials (Table 1). Based on the opinions of Chinese ophthalmologists, it was assumed that PDT was administered to all AMD patients regardless of lesion subtype for ethical reasons, although PDT is indicated in patients with predominantly classic lesions. The VIP and TAP trials reported the percentages of patients experiencing a 3-line gain, 3- to 6-line loss, and >6-line loss among patients receiving PDT with verteporfin and a placebo (usual care) at 1 and 2 years [9, 26–29]. An exponential distribution was used for the 3-month transition probabilities between health states. Thus, the 3-month transition probabilities could be calculated by the density method [30]. To extrapolate effects beyond the time horizon of the trials, estimated transition probabilities for year 2 in the usual care cohort were applied to each state of the model from year 3 until death. The MARINA trial compared the efficacy of ranibizumab to usual care in patients with minimally classic and occult lesions and the ANCHOR trial made a comparison between ranibizumab and PDT for predominantly classic lesions [11, 14]. Due to a lack of published studies directly comparing the four treatment strategies, an indirect comparison was performed using the risk ratio in cumulative probabilities between ranibizumab and PDT or usual care to project the model output. The results of the CATT trial were used to estimate the RR between bevacizumab and ranibizumab for the three types of lesions [13].

### 2.3. Resource Utilization and Costs

This analysis was conducted from the perspective of the Chinese health care system. Costs are presented in 2012 US dollars ($). Direct medical costs were incorporated into the model, including costs related to AMD treatment and follow-up, direct medical costs related to AMD comorbidities, and direct nonmedical-related costs. All of the health resource unit costs were estimated using data from local health systems or the National Development and Reform Commission (NDRC) of China [31].

With regard to medical costs associated with AMD treatment, unit costs for physician consultations and diagnostic procedures were estimated from a previous study [32]. The model assumed that patients in the usual care arm would receive routine follow-up and supportive care but not active treatment. The average number of treatments with PDT in years 1 and 2 was obtained from the VISION trial, which showed that patients receiving treatment with PDT at the physician's discretion had 2.05 and 1.54 drug administrations in years 1 and 2, respectively [12]. Ranibizumab was administered at a dosage of 0.5 mg as an outpatient procedure. The frequency of injections was eight in the first year and six in the second year, which was derived from data obtained in the ANCHOR and MARINA trials [11, 14]. For bevacizumab, the administered dosage was 1.25 mg and the frequency of injections was assumed to be similar to ranibizumab per their equivalent effects on visual acuity when administered according to the same schedule [13]. The annual mean cost associated with PDT, bevacizumab, and ranibizumab treatment in these arms was measured by multiplying the average frequency of treatments per year by the percentage of patients receiving the treatment and by the average cost of treatment. Age-specific natural mortality rates were obtained from the life tables for WHO member states (2011) [33].

The unit costs of managing SAEs (e.g., endophthalmitis, traumatic injury to the lens, and retinal detachment) were estimated based on the treatment path recommended by a panel of Chinese clinical ophthalmologists. The rates of SAEs were obtained from the clinical trials (Table 2).

Low vision increases the risks of numerous comorbidities, such as depression, falls, and the need for assisted living [9, 34]. The model estimated the costs associated with comorbidities for patients with varying degrees of visual acuity. The prevalence of depression in China was derived from a community-based study reported by Chen et al. [35]. The incidence of a one-time fall is nearly 18% according to the literature [36]. Based on interviews with clinical experts, the probability of receiving assisted living services was assumed to be similar to that in the report published by Earnshaw et al. [9]. Sensitivity analysis was performed to examine the impact of this assumption. The unit costs associated with comorbidities are depicted in Table 2.

### 2.4. Health Utilities

Due to the absence of reported Chinese-specific utility scores as to any degree of VA impairment, the utility values of the five VA states were derived from the study reported by Brown et al. using the time-tradeoff method [37] and it is assumed that they also represent local patients (Table 3). In the current analysis, only the bilateral disease treatment in the better-seeing eye was evaluated because there is a more notable correlation between utility values and VA for the better-seeing eye than for the poorer-seeing eye [37].

### 2.5. Sensitivity Analyses

Sensitivity analyses are typically conducted to test whether a model has any structural errors (i.e., to ensure that it is robust) and to assess how outcomes are substantially affected when specific parameters are changed. One-way sensitivity analyses were conducted to examine the impact of input parameters in the model on the robustness of results over the ranges shown in Tables 1–3 or assuming ±20% of mean values. A probabilistic sensitivity analysis (second-order Monte Carlo simulation) was conducted using a cohort of 1000 simulations to simultaneously examine the impact of uncertainty across all parameters. Proportions, probabilities, and utilities were subject to beta distribution, whereas cost was sampled from lognormal distribution with an assumed standard deviation of 20% from mean values. The results are shown on a cost-effectiveness plane. The outcomes projected from all 1000 simulations were used to plot acceptability curves, which estimated the willingness to pay (WTP) threshold for an incremental unit of effectiveness.

## 3. Results

### 3.1. Base-Case Analysis

In the base-case analysis (Table 4), treating AMD patients with ranibizumab, compared to the other three alternatives, provided the greatest clinical outcomes at a higher cost for all types of lesions in the lifetime horizon. In patients with predominantly classical, minimally classical, and occult types of lesions, the estimated lifetime costs for the ranibizumab arm were notably 240% higher than usual care, 61% higher than PDT, and 219% higher than bevacizumab; the respective projected marginal gains in QALYs for patients with predominantly classical, minimally classical, and occult types of lesions were 15%, 5%, and 9% for usual care; 9%, 3%, and 6% for PDT; and 2%, 1%, and 1% for bevacizumab. PDT was less cost-effective than bevacizumab and was dominated due to fewer health benefits and higher costs regardless of lesion type. The ICERs of bevacizumab over usual care ranged from $1,258 for predominantly classical to $3,803 for minimally classical, which were lower than the two other active treatment alternatives.

### 3.2. Sensitivity Analysis

**Figure 2:**
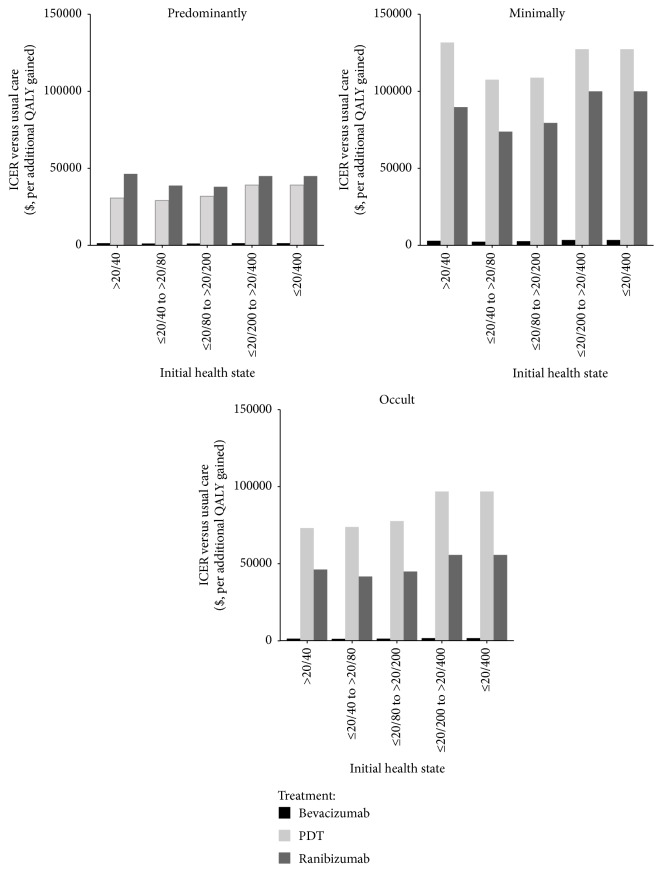
Sensitivity analysis of the effects of the initial health states. Values are indicated (*y*-axis) as cost per additional quality-adjusted life-year (QALY) gained.

The one-way sensitivity analyses showed that some model variables had a substantial impact on the results, which are presented in Table 5. The substantially influential variables included age, utility values, RR of bevacizumab over ranibizumab, frequency of bevacizumab injections, and the cost of bevacizumab and intravitreal injection. Other parameters, such as the cost and probability of SAEs, produced little model output sensitivity. One-way sensitivity analyses also showed that the ICERs of active treatment were more favorable in patients with VA ≤20/40 to >20/80 for all three types of lesions (Figure 2).

**Figure 3:**
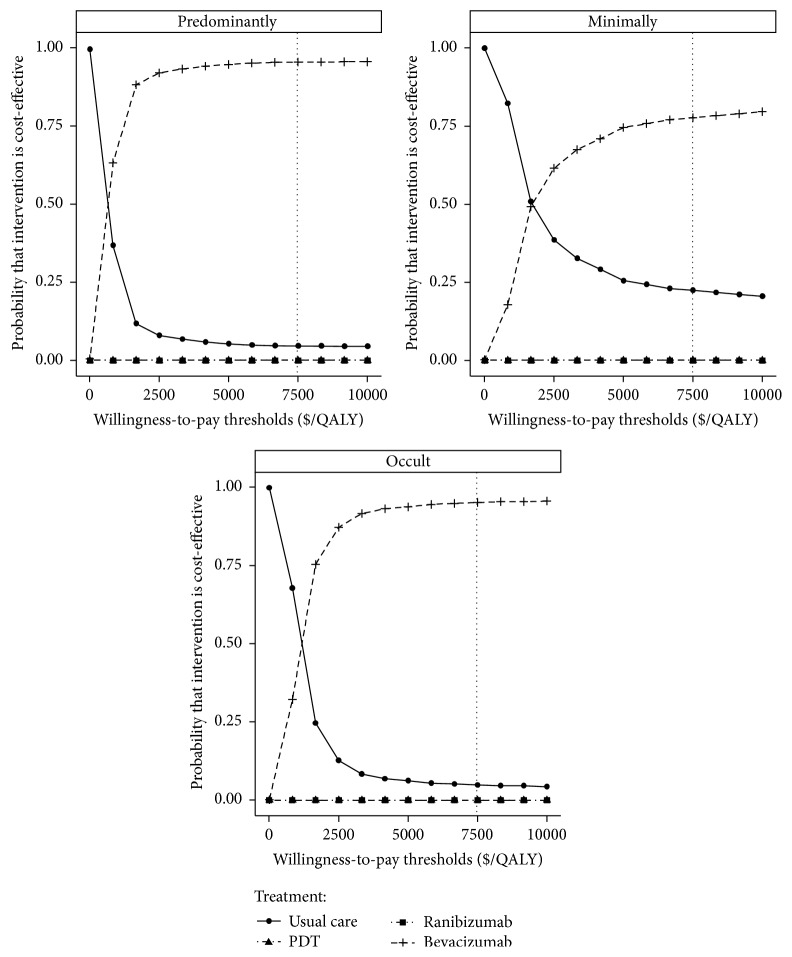
The cost-effectiveness acceptability curves for the four treatment strategies. The *y*-axis indicates the probability that a strategy is cost-effective against the willingness to pay per QALY gained (*x*-axis). The vertical dotted line represents the thresholds for China.

Pursuant to a probabilistic sensitivity analysis, acceptability curves showed that AMD treatment with bevacizumab, compared to usual care, PDT, and bevacizumab, yielded acceptable ICERs in most cases at the $7,480/QALY threshold for all patients, regardless of lesion type (Figure 3).

## 4. Discussion

There was great excitement among ophthalmologists and patients after clinical trials demonstrated the medical benefits of ranibizumab and bevacizumab. However, in the context of limited health care resources, the widespread use of new drugs, especially those that are more effective but more expensive than the competing alternatives, would increase the socioeconomic burden on people and societies. Thus, the economic evaluation of ranibizumab and bevacizumab in the clinical setting is urgently needed. In this light, we conducted a cost-effectiveness analysis of three active treatments for patients with AMD in the Chinese health care setting: bevacizumab, ranibizumab, and verteporfin with PDT versus usual care.

Results of the analysis indicated that the cost per QALY gained with bevacizumab therapy over usual care in a lifetime horizon was $1,258, $3,803, and $2,066 for predominantly classical, minimally classical, and occult lesions, respectively, just below the per capita GDP of China ($7,480 in 2011), which is highly cost-effective according to WHO recommendations [24, 25]. Another anti-VEGF drug, ranibizumab, yielded the greatest clinical outcomes compared to the alternatives under examination, but at a higher cost. The ICERs of ranibizumab over usual care were all greater than three times the per capita GDP of China, which indicates that ranibizumab is not a cost-effective option in the Chinese setting. Verteporfin with PDT was dominated by bevacizumab due to its lower health outcomes and higher cost. This outcome, however, was affected by alternative scenarios explored in the sensitivity analyses. They corroborated calculations that showed that the administration of bevacizumab was cost-effective in nearly 95.4%, 77.6%, and 95.2% of predominantly classical, minimally classical, and occult lesion cases, respectively. In addition to a probabilistic sensitivity analysis, multiple one-way sensitivity analyses were performed to test the robustness of the model. The results of the majority of these analyses did result in the ICERs of bevacizumab exceeding the $7,480 threshold; thus, the robustness of the main analysis was further strengthened (Table 5). The time horizon of the analysis had a substantial impact on model output, partly because short time horizons could not reflect subsequent long-term health benefits.

This study is one of the few economic analyses to compare costs and health outcomes of bevacizumab, ranibizumab, and verteporfin with PDT. Our conclusion was similar to the report recently published by Patel et al. [38]. The authors evaluated the cost-effectiveness of bevacizumab relative to ranibizumab for AMD based on a four-state Markov model for 20 years. They found that the ICER for bevacizumab was dominant compared with ranibizumab. However, different types of lesions were not taken into account in their analysis; the ICER of bevacizumab was −$54,649 over ranibizumab per marginal QALY because bevacizumab yielded 3.48 more QALYs than ranibizumab, and the cost for bevacizumab treatment was $190,300 lower than ranibizumab. These findings were notably different than our results. In our analysis, treatment with ranibizumab produced more health outcomes than bevacizumab because the mean change in visual acuity score favored treatment with ranibizumab, although no statistical analysis was detected according to the results of the CATT trial [13]. One of the potential reasons for this difference is that the study reported by Patel et al. used a different model design and did not adjust the data from varied sources before incorporating it into the model. In addition to the comparison between bevacizumab and ranibizumab, several published studies have evaluated the cost-effectiveness of ranibizumab relative to PDT and usual care [20, 39, 40]. These studies found that ranibizumab can be a cost-effective option in comparison with selected alternatives because the ICERs were relatively lower than the corresponding threshold recommended by their local health care systems. It should be noted that these studies were conducted with regard to developed countries, which had relatively rich health resources and a higher threshold than developing countries, such as China. To the best of our knowledge, our analysis is the first economic investigation associated with the treatment of AMD. Our findings might supply some reference information for patients, physicians, and decision-makers from developing regions.

The results of this analysis also showed that early treatment was accompanied by improved clinical outcomes at a lower cost with respect to all three types of lesions and the greatest incremental benefits were observed in the health state with VA ≤20/40 to >20/80 (Figure 2). This result parallels the findings of a cost-effective analysis reported by Javitt et al., which showed that treatment with pegaptanib should be started as early as possible to maximize clinical and economic benefits [41]. In addition to the early VA health state, results from one-way sensitivity analysis found that younger patients could achieve more favorable economic outcomes in cases of predominantly classical and occult lesions (Table 3). When a patient with VA ≤20/40 to >20/80 was diagnosed with AMD at 50 years old, the ICERs of bevacizumab therapy over usual care fell to $1,148 and $2,032 for predominantly classical and occult lesions, respectively; when a patient was 88 years old, the ICERs increased to $1,774 and $2,972, respectively. These findings suggest that early community screening and intervention may be attractive. Recently, a system for automated detection of early AMD signs from retinal photographs was reported, with sensitivity and specificity rates of 75% [42]. Such progress makes the early detection of AMD more feasible.

Similar to all modeling analyses, our study had several limitations that require consideration. First, the current analysis used an indirect method to evaluate the health and economic outcomes of the four treatments due to the absences of direct head-to-head studies comparing bevacizumab, ranibizumab, and verteporfin with PDT with usual care. This would lead to inevitable uncertainty in the results because the data from the varied studies had a high degree of heterogeneity, given the different study designs, patient cohorts, and dosing schedules. To examine the robustness of the model, sensitivity analyses were conducted by varying model parameters. Future analyses should be performed if the data from head-to-head trials become available. Second, due to the unavailability of Chinese-specific data, especially efficacy and utility data, the current analysis used data mainly obtained from literature published abroad, which would produce a degree of uncertainty with respect to Chinese data, pursuant to the hypothesis that foreign observations are similar to those in a Chinese setting. However, one recent study showed that the efficacy of bevacizumab outcomes compared favorably to a Chinese population, which would result in the underestimation of the cost-effectiveness of bevacizumab [5]; moreover, as suggested by some Chinese ophthalmologists, the quality of life of AMD patients in China should not be significantly different from that of external AMD patients. Although efficacy and utilities had some impact on our results, the results of the sensitivity analysis indicated that their influence was limited. Third, we did not examine the “continuous treatment effect” approach under which active treatments, especially the bevacizumab and ranibizumab treatments, would be administered beyond the trial time frames because the efficacy of continuous treatment beyond 2 years is still unclear. Fourth, this study excluded indirect costs, such as the loss of productivity, which may have resulted in a substantial social economic burden for a patient's family and society. If these indirect costs were considered, this analysis would have underestimated the cost-effectiveness of active treatment, which would become more cost-effective because indirect costs would be decreased by active treatments. Finally, to simplify the model, utility decrements for adverse events were not taken into account because the adverse events of bevacizumab, ranibizumab, and verteporfin with PDT were both mild and infrequent; this had little impact on the outcomes as depicted by Brown et al. [43]. Nevertheless, we are confident that the model faithfully represented the common clinical conditions of AMD in a health resource-limited setting. We believe that this study has the potential to be an important reference point for decision-makers.

In conclusion, although bevacizumab is not licensed for the treatment of AMD in China, bevacizumab is highly cost-effective compared with ranibizumab and verteporfin with PDT because of the more favorable ICER in the Chinese health care setting. To reduce the financial burden related to AMD, we suggest that the government license off-label use of bevacizumab and set strict rules for avoiding sterile endophthalmitis [44, 45]. Early intervention would improve the cost-effectiveness of active treatments.

## Figures and Tables

**Table 1:** Cumulative probability and RR of losing or gaining visual acuity.

Parameters	Predominantly classic	Minimally classic	Occult with no classic
Cumulative probability of change in vision			
Usual care at 1 year			
Gain of >3 lines	2.41%	1.92%	2.17%
Loss of 3–6 lines	23.10%	28.85%	22.83%
Loss of >6 lines	29.14%	16.35%	32.61%
Usual care at 2 years			
Gain of >3 lines	1.48%	2.89%	1.71%
Loss of 3–6 lines	6.33%	6.33%	6.33%
Loss of >6 lines	4.21%	10.57%	14.13%
PDT at 1 year			
Gain of >3 lines	5.66%	6.44%	3.01%
Loss of 3–6 lines	26.51%	27.23%	28.92%
Loss of >6 lines	13.46%	16.83%	22.29%
PDT at 2 years			
Gain of >3 lines	1.75%	1.98%	1.81%
Loss of 3–6 lines	6.35%	5.44%	8.16%
Loss of >6 lines	3.00%	2.97%	6.63%
RR of ranibizumab versus PDT			
at 1 year			
Gain of >3 lines	7.2	N/A	N/A
Loss of 3–6 lines	0.1	N/A	N/A
Loss of >6 lines	0	N/A	N/A
at 2 years			
Gain of >3 lines	0.49	N/A	N/A
Loss of 3–6 lines	4.35	N/A	N/A
Loss of >6 lines	0.06	N/A	N/A
RR of ranibizumab versus usual care			
at 1 year			
Gain of >3 lines	N/A	6.69	6.69
Loss of 3–6 lines	N/A	0.17	0.17
Loss of >6 lines	N/A	0.09	0.09
at 2 years			
Gain of >3 lines	N/A	0.42	0.42
Loss of 3–6 lines	N/A	3.78	3.78
Loss of >6 lines	N/A	0.14	0.14
RR of bevacizumab versus ranibizumab			
at 1 and 2 years			
Gain of >3 lines	0.92	0.92	0.92
Loss of 3–6 lines	1.07	1.07	1.07
Loss of >6 lines	1.07	1.07	1.07

RR: risk ratio.

N/A: not applicable.

**Table 2:** Direct costs^*∗*^ related to AMD treatment and comorbidities.

Parameters	Median unit cost (range)	Average number (range)	Annual mean total cost	Source
Ophthalmologist consultation	3.2 (0.8–47.6)	1 time per cycle	12.8	[32]
Optical coherence tomography	31.7 (23.8–39.7)	1 time per cycle	126.8	[32]
Fluorescein angiography	58.7 (44–73.4)	2 times per year	234.8	[32]
Verteporfin per 15 mg	2539.7 (1904.8–3174.6)^#^			[32]
Laser activation	238.1 (158.7–317.5)			[32]
PDT (verteporfin per 15 mg + laser activation)		2.05 (1.5375–2.5625) at 1 year	5694.5 at 1 year	Calculated
		1.54 (1.155–1.925) at 2 years	4277.8 at 2 years	Calculated
Intravitreal injection	41.3 (31–51.6)			Local charge
Ranibizumab per 0.5 mg	1523.8 (1142.9–1904.8)^#^	8 (6–12) at 1 year	12190.4 at 1 year	Local charge
		6 (4.5–12) at 2 years	9142.8 at 2 years	
Bevacizumab per 1.25 mg	10.5 (7.9–13.1)^#^	8 (6–12) at 1 year	84 at 1 year	Local charge
		6 (4.5–12) at 2 years	63 at 2 years	
Adverse reactions				
Endophthalmitis				Local charge
Ranibizumab arm		1.4% (1.05%–1.75%) during 1 year	22.2	
Bevacizumab arm		2.8% (2.1%–3.5%) during 1 year	44.4	
Lens damage				Local charge
Ranibizumab arm		0.4% (0.3%–0.5%) during 1 year	6.3	
Bevacizumab arm		0.4% (0.3%–0.5%) during 1 year	6.3	
Retinal detachment				Local charge
Ranibizumab arm		0.3% (0.23%–0.38%) during 1 year	5.7	
Bevacizumab arm		0.3% (0.23%–0.38%) during 1 year	5.7	
Comorbidities				
Depression	130.6 (111.2–156.4)	2.2% (1.65%–2.75%) during 1 year	3	Local charge
Fall	1093.9 (364.6–1823.2)	18% (14.50%–28.60%) during 1 year	23	Local charge
Assisted living	432.7 (324.5–540.8)	2.1% (1.58%–2.63%) during 1 year	77.9	Local charge

^*∗*^Costs are presented as US dollars (January 2015 exchange rate, US$ = CYN 6.30).

^#^The range was used for sensitivity analysis.

**Table 3:** Utilities for each visual acuity state.

Visual acuity	Utility value
>20/40	0.89 (0.82–0.96)
≤20/40 to >20/80	0.81 (0.73–0.89)
≤20/80 to >20/200	0.57 (0.47–0.67)
≤20/200 to >20/400	0.52 (0.38–0.66)
≤20/400	0.40 (0.29–0.50)

**Table 4:** Lifetime results for the reference case.

Treatment arms	Costs ($)	Vision-years	QALYs	ICER versus usual care	Comments
Predominantly classic					
Usual care	8,618.5	2.38	3.97		
PDT	18,292.5	3.23	4.19	44,333	Dominated
Ranibizumab	29,468.3	4.16	4.55	36,089	Not cost-effective
Bevacizumab	9,232.8	3.92	4.46	1,258	Cost-effective
Minimally classic					
Usual care	8,663.5	2.80	4.10		
PDT	18,289.1	3.11	4.19	112,992	Dominated
Ranibizumab	29,480.0	3.77	4.31	102,828	Not cost-effective
Bevacizumab	9,242.8	3.59	4.26	3,803	Cost-effective
Occult with no classic					
Usual care	8,594.9	2.08	3.90		
PDT	18,240.1	2.43	4.01	91,424	Dominated
Ranibizumab	29,465.1	3.61	4.26	58,790	Not cost-effective
Bevacizumab	9,227.8	3.44	4.21	2,066	Cost-effective

**Table 5:** One-way sensitivity results of bevacizumab.

Parameters	ICER (versus usual care)		
	Predominantly	Minimally	Occult
Age			
55	1,148	11,278	2,032
88	1,774	4,368	2,972
RR (bevacizumab versus ranibizumab)			
Gain of >3 lines at 1 year			
Base − 20%	1,604	4,700	2,308
Base + 20%	1,023	3,183	1,883
Loss of 3–6 lines at 1 year			
Base − 20%	1,256	3,677	2,052
Base + 20%	1,272	3,937	2,101
Loss of >6 lines at 1 year			
Base − 20%	1,263	3,579	1,982
Base + 20%	1,265	4,052	2,177
Gain of >3 lines at 2 years			
Base − 20%	1,264	3,802	2,076
Base + 20%	1,264	3,802	2,076
Loss of 3–6 lines at 2 years			
Base − 20%	975	2,142	1,572
Base + 20%	1,708	12,922	2,921
Loss of >6 lines at 2 years			
Base − 20%	1,264	3,802	2,076
Base + 20%	1,264	3,802	2,076
Utility			
>20/40			
0.82	1,296	3,841	2,088
0.96	1,234	3,763	2,064
≤20/40 to >20/80			
0.73	1,477	4,364	2,271
0.89	1,104	3,368	1,912
≤20/80 to >20/200			
0.47	1,405	5,762	3,088
0.67	1,149	2,837	1,563
≤20/200 to >20/400			
0.38	1,155	2,791	1,984
0.66	1,396	5,961	2,177
≤20/400			
0.29	993	2,963	1,430
0.5	1,681	5,118	3,523
Cost ($)			
Bevacizumab per 1.25 mg			
7.9	1,191	3,571	1,960
13.1	1,336	4,033	2,191
Intravitreal injection			
31	1,102	3,287	1,818
51.6	1,426	4,317	2,334
Frequency of bevacizumab injections at 1 year			
6	1,221	3,666	2,008
12	1,349	4,074	2,212
Frequency of bevacizumab injections at 2 years			
4.5	1,234	3,707	2,029
12	1,383	4,181	2,266
Time horizon			
2 years	3,935	8,498	7,227
10 years	1,277	3,353	2,064
